# Fermented Foods, Functional Nutrition, and Maternal Gut Microbiota During Pregnancy: Molecular Mechanisms and the Maternal–Infant Microbiome Axis

**DOI:** 10.3390/ijms27146488

**Published:** 2026-07-21

**Authors:** Tuğba Küçükkasap, Asmin Yavuz, Özde Kuran

**Affiliations:** 1 Department of Nutrition and Dietetics, Gülhane Health Sciences Faculty, University of Health Sciences, 06018 Ankara, Turkey; 2 Department of Nutrition and Dietetics, Gülhane Health Sciences Institute, University of Health Sciences, 06018 Ankara, Turkey; asminyavuz29@gmail.com (A.Y.); ozdekuran@gmail.com (Ö.K.)

**Keywords:** pregnancy, maternal gut microbiota, fermented foods, intestinal barrier function, maternal–fetal programming

## Abstract

Pregnancy is associated with profound metabolic, hormonal, and immunological adaptations accompanied by dynamic alterations in maternal gut microbiota composition and function. Emerging evidence suggests that maternal diet is a major regulator of these microbiota-related changes and may influence maternal–fetal health through microbial metabolites and host signaling pathways. Fermented foods and functional dietary components, including prebiotics, probiotics, synbiotics, and polyphenols, have gained increasing attention because of their potential to modulate gut microbial diversity, intestinal barrier integrity, inflammatory responses, and metabolic homeostasis. Mechanistically, these effects are mediated through pathways involving short-chain fatty acids, G protein-coupled receptors, nuclear factor kappa B signaling, histone deacetylase inhibition, and immune cell regulation. Altered microbiota-associated signaling has been linked to gestational metabolic disorders such as obesity, gestational diabetes mellitus, and preeclampsia, as well as fetal immune and metabolic programming. Particular emphasis is placed on the maternal–infant microbiome axis, highlighting how maternal nutrition and microbiota-mediated signaling may influence microbial transmission, fetal programming, and early-life microbiome development. This review summarizes current evidence regarding pregnancy-associated gut microbiota alterations and discusses the molecular mechanisms through which fermented foods and functional nutrition may influence maternal and fetal health outcomes.

## 1. Introduction

### 1.1. Diet–Microbiota Interactions During Pregnancy

Pregnancy is characterized by profound endocrine, metabolic, and immunological adaptations accompanied by dynamic alterations in maternal gut microbiota composition and function [1,2]. These physiological microbial adaptations are considered an integral component of healthy pregnancy and support maternal metabolic adaptation, immune tolerance, and fetal development before any pregnancy-related pathological conditions arise [1,3,4].

During pregnancy, maternal gut microbiota undergoes substantial remodeling in response to hormonal changes, increased energy demands, immune adaptations, and environmental factors. Late pregnancy is commonly associated with reduced microbial diversity and increased abundance of Proteobacteria and certain Firmicutes taxa, which contributes to physiological insulin resistance and metabolic adaptations supporting fetal growth [1,2]. However, maternal obesity, high-fat diets, low dietary fiber intake, and poor nutritional status may promote gut dysbiosis and contribute to pregnancy-related complications including gestational diabetes mellitus, preeclampsia, and preterm birth [2,5].

The term gut microbiota refers to the community of microorganisms, including bacteria, archaea, viruses, and fungi, that inhabit the gastrointestinal tract [1]. Although the gut microbiota comprises all these microbial groups, this review primarily focuses on bacterial communities because current evidence regarding maternal diet, fermented foods, and microbiota-mediated mechanisms during pregnancy is predominantly based on bacterial taxa and their metabolites [1,6].

Stool sampling is useful in human studies because of its accessibility, but it represents only a proxy for the intestinal microbiota and does not fully reflect the spatial and functional diversity of microbial communities throughout the gastrointestinal tract [7].

The relationship between maternal diet and gut microbiota is bidirectional and highly dynamic. Dietary components influence microbial composition and metabolic activity, whereas gut microorganisms transform nutrients into bioactive metabolites that affect host physiology. In particular, fiber-rich and prebiotic-containing dietary patterns have been associated with increased abundance of *Bifidobacterium* and butyrate-producing bacteria, while Western-style diets characterized by high fat and low fiber intake may favor dysbiosis and proinflammatory microbial profiles [2,6].

Microbiota-derived metabolites, especially short-chain fatty acids (SCFAs), may also influence maternal–fetal communication through placental signaling and, potentially, transplacental transfer, thereby affecting fetal immune, metabolic, and neurodevelopmental processes [3,8]. Consequently, maternal nutrition and microbiota interactions are increasingly considered central components of early-life programming within the “first 1000 days” framework [9,10].

Growing evidence suggests that microbiota-targeted nutritional strategies have been proposed to support maternal and fetal health. In this review, the term functional dietary components refers to food-derived constituents with documented microbiota-modulating properties, including prebiotics, probiotics, synbiotics, and polyphenols. Together with fermented foods, these dietary components have attracted increasing attention because of their potential roles in regulating microbial diversity, intestinal barrier integrity, inflammatory responses, and metabolic homeostasis [6,9,11,12,13].

Fermented foods and functional dietary components may influence maternal gut microbiota through several complementary mechanisms. Fermented foods provide live microorganisms and fermentation-derived bioactive metabolites, whereas prebiotics, probiotics, synbiotics, and polyphenols modulate microbial composition, stimulate short-chain fatty acid production, reinforce intestinal barrier integrity, and regulate immune and metabolic signaling pathways. These microbiota-mediated mechanisms provide the conceptual basis for the present review and are discussed in detail in the following sections [6,12,13,14,15,16].

Pregnancy represents a critical window during which maternal nutrition is one of the few modifiable factors capable of influencing both maternal physiology and fetal development [6,8,9,10]. Among dietary strategies, fermented foods and functional dietary components have attracted increasing scientific interest because they not only provide essential nutrients but also contain live microorganisms, microbiota-accessible substrates, and bioactive compounds that can modulate gut microbial composition and function [13,14,15,17]. Although these foods are not considered essential dietary components during pregnancy, accumulating evidence suggests that they may contribute to intestinal barrier integrity, immune regulation, microbial metabolite production, and metabolic homeostasis through microbiota-mediated mechanisms [6,12,16]. Therefore, this review specifically focuses on fermented foods and functional nutrition as promising microbiota-targeted nutritional approaches that appear to contribute to maternal and offspring health.

### 1.2. Pregnancy-Induced Alterations in Gut Microbiota

During healthy pregnancy, the maternal gut microbiota undergoes compositional and functional alterations that support both maternal and fetal physiology [10]. In the first trimester, the gut microbiota profile largely resembles that of healthy non-pregnant individuals, with butyrate-producing bacteria such as *Clostridiales* and *Faecalibacterium prausnitzii* being predominant. As pregnancy progresses, substantial microbial restructuring occurs; α-diversity decreases while β-diversity increases between the second and third trimesters [18]. These changes are considered part of the physiological metabolic adaptations that support fetal growth and increased energy demands. Rather than representing dysbiosis, many of these changes are considered physiological adaptations that facilitate the increased metabolic and immunological demands of pregnancy and support normal fetal growth [15,17,18].

In late pregnancy, the abundance of anti-inflammatory butyrate-producing bacteria declines, whereas the relative abundance of Proteobacteria, *Bifidobacterium*, and lactic acid-producing bacteria increases [18,19,20]. Parallel to these microbial shifts, metabolic alterations associated with low-grade inflammation, physiological insulin resistance, and enhanced energy storage become more pronounced. In overweight and obese pregnant women, an increased Firmicutes–Bacteroidetes ratio has been reported [21].

Throughout pregnancy, the gut microbiota is reshaped toward microbial profiles that enhance energy extraction and substrate utilization efficiency. Alterations in *Akkermansia*, *Bifidobacterium*, and certain members of the Firmicutes phylum may be associated with energy homeostasis and fat storage, whereas increases in Actinobacteria and Proteobacteria may contribute to protection of the maternal–fetal unit against infections [20,22,23,24].

Although these microbial changes are largely physiological during healthy pregnancy, excessive deviations associated with maternal obesity or other metabolic disturbances may lead to gut dysbiosis and contribute to pregnancy-related complications. However, reductions in butyrate-producing bacteria such as *Faecalibacterium prausnitzii* and decreased SCFA synthesis during the third trimester have been associated with low-grade inflammation, reduced insulin sensitivity, and increased intestinal energy absorption [18,19,20]. These alterations, particularly in the presence of maternal obesity and gestational metabolic disorders, suggest that gut dysbiosis may exacerbate metabolic inflammation during pregnancy [18,22,25].

Pregnancy-associated physiological and pathological alterations in gut microbiota composition, microbial metabolites, and related metabolic outcomes are summarized in Table 1.

Therefore, this review discusses fermented foods as a food-based dietary approach and functional dietary components as specific microbiota-targeted nutritional interventions [12,13,14,15,17].

### 1.3. Literature Search Strategy

Relevant literature published in English was identified through systematic searches of PubMed, Scopus, and Web of Science databases. The search strategy combined controlled vocabulary and free-text terms related to pregnancy, gut microbiota, fermented foods, and microbiota-targeted nutritional interventions. The PubMed search strategy included the following search string: (“pregnancy” OR “pregnant women”) AND (“gut microbiota” OR microbiome) AND (“fermented foods” OR probiotics OR prebiotics OR synbiotics OR polyphenols OR “functional nutrition”). Similar search strategies adapted to the indexing terms of Scopus and Web of Science were applied. Detailed search strategies for each database are provided in Supplementary Table S1.

Priority was given to recent mechanistic studies, randomized controlled trials, systematic reviews, and meta-analyses published within the last 10 years. Additional landmark studies and relevant experimental animal studies investigating microbiota-mediated molecular mechanisms were also included.

Eligible publications included original human studies, randomized controlled trials, systematic reviews, meta-analyses, and relevant mechanistic animal studies published in English. Conference abstracts, editorials, letters, duplicate publications, non-English articles, and studies not directly related to maternal gut microbiota, fermented foods, functional nutrition, or pregnancy were excluded. Studies were selected based on their relevance to molecular mechanisms, translational significance, and maternal–fetal microbiota-related outcomes.

Where human pregnancy data were limited, findings from non-pregnant populations, animal models, and in vitro studies were included to provide mechanistic insights and should be interpreted as hypothesis-generating rather than direct evidence for clinical effects during pregnancy.

## 2. Molecular Basis of Gut Microbiota–Host Interactions

Gut microbiota–host interactions during pregnancy extend beyond alterations in microbial composition and involve a complex regulatory network including microbial metabolites, receptor-mediated signaling pathways, and immune responses [1,31]. These interactions play a central role in maternal metabolic adaptation, immune tolerance, and fetal development [25,32].

Although many microbiota–host signaling pathways are not unique to pregnancy, gestation is characterized by profound hormonal, metabolic, and immunological adaptations that remodel these pathways. Pregnancy-specific alterations in microbial composition and metabolite production may modify the activity of signaling pathways involved in immune tolerance, metabolic adaptation, and maternal–fetal communication, thereby distinguishing gestational microbiota–host interactions from those observed in non-pregnant individuals [1,2,3].

Short-chain fatty acids (SCFAs), including acetate, propionate, and butyrate, are among the key mediators of microbiota–host signaling. Produced through microbial fermentation of dietary fiber, SCFAs exert their effects primarily via G protein-coupled receptors such as GPR41, GPR43, and GPR109A. Activation of these pathways modulates inflammatory and metabolic signaling, suppresses NF-κB activation, and reduces proinflammatory cytokine production. In addition, butyrate functions as a histone deacetylase (HDAC) inhibitor, thereby regulating gene expression through epigenetic mechanisms and promoting anti-inflammatory responses [1,25]. Altered SCFA metabolism during late pregnancy has been associated with physiological low-grade inflammation and reduced insulin sensitivity [18,25].

Another important mechanism involves recognition of microbial components by the host immune system. Lipopolysaccharides (LPS) derived from Gram-negative bacteria activate Toll-like receptor 4 (TLR4)-mediated MyD88 signaling pathways, leading to increased production of proinflammatory cytokines such as TNF-α, IL-6, and IL-1β through NF-κB activation. Increased abundance of Proteobacteria during pregnancy may contribute to elevated LPS levels, metabolic endotoxemia, and systemic inflammation [1].

Gut microbiota also contributes to the maintenance of immune tolerance. SCFAs promote the proliferation and function of regulatory T cells (Tregs) and enhance the production of anti-inflammatory cytokines including IL-10 and TGF-β [33,34]. These mechanisms are considered important for the development of maternal immune tolerance toward the fetus. Additionally, acetate may support Treg activation through increased expression of the autoimmune regulator gene (AIRE) [34].

Microbiota-derived metabolites and microbial products may also influence the fetoplacental unit. SCFAs, LPS, peptidoglycans, and other microbial derivatives can potentially cross the placental barrier via syncytiotrophoblast-mediated mechanisms and affect fetal immune development [35,36].

Better understanding of these mechanisms may contribute to the development of microbiota-targeted strategies for preventing pregnancy-related complications [37,38,39]. Overall, these signaling pathways are not unique to pregnancy; however, their regulation is dynamically remodeled throughout gestation in response to physiological endocrine and metabolic adaptations [1,2].

## 3. Dietary Regulation of Gut Microbiota: A Molecular Perspective

Following the overview of physiological gut microbiota adaptations during pregnancy, this section focuses on the molecular mechanisms through which maternal diet is thought to regulate gut microbiota composition and function.

Alterations in gut microbiota during pregnancy are influenced not only by hormonal and metabolic signals but also significantly regulated by maternal diet. Both animal and human studies have demonstrated that dietary components can modify gut microbiota composition, microbial metabolite production, and host metabolic responses [33,40].

High-fat diets during pregnancy have been associated with substantial alterations in gut microbial composition, affecting metabolic pathways related to lipid metabolism, glycolysis, and inflammation, thereby influencing intestinal barrier function and immune responses [33,40]. Human studies further suggest that maternal obesity and excessive gestational weight gain are associated with microbial shifts involving Bacteroides, Staphylococcus, and certain members of the Proteobacteria phylum [22].

Macronutrient distribution also appears to shape pregnancy-associated microbiota profiles. High carbohydrate intake has been linked to increased abundance of Proteobacteria and Bacteroides, along with reductions in some butyrate-producing Firmicutes species [41]. Similarly, high-fat diets may decrease beneficial bacteria such as Lachnospira and Ruminococcus, potentially impairing intestinal barrier integrity [42]. Dietary protein sources may also influence microbial composition, with animal protein-rich diets being associated with alterations in microbial metabolites and bacterial subgroups [43].

Maternal metabolic status may further modulate diet–microbiota interactions. Certain microbiota changes associated with polyunsaturated fatty acid (PUFA) intake have been observed predominantly in normal-weight pregnant women, suggesting that microbial responses to dietary components may vary according to maternal metabolic profile [6,44].

In contrast, fiber-rich diets, prebiotics, and Mediterranean-style dietary patterns have consistently been associated with increased microbial diversity and enhanced short-chain fatty acid (SCFA) production [43,44,45,46,47]. Fiber-rich dietary patterns have been associated with increased abundance of butyrate-producing bacteria including *Roseburia* and Lachnospiraceae [40,41]. Additionally, dietary fiber and omega-3 fatty acid intake have been associated with lower zonulin levels [48].

**Figure 1:**
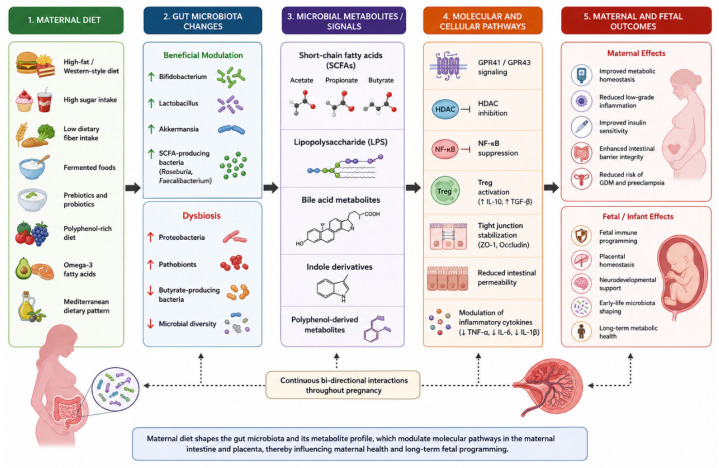
Mechanistic links between maternal diet, gut microbiota, and fetal programming during pregnancy. Maternal dietary patterns, including fermented foods and functional dietary components, modulate gut microbial composition and metabolite production, particularly short-chain fatty acids (SCFAs). These microbial metabolites influence intestinal barrier integrity, immune regulation, and metabolic homeostasis through G protein-coupled receptor (GPR41/GPR43) signaling, nuclear factor kappa B (NF-κB) modulation, and histone deacetylase (HDAC) inhibition, ultimately contributing to maternal–fetal communication and fetal programming. Abbreviations: SCFAs, short-chain fatty acids; GPR, G protein-coupled receptor; NF-κB, nuclear factor kappa B; HDAC, histone deacetylase. Upward and downward arrows indicate increases and decreases, respectively, whereas dashed bidirectional arrows represent continuous interactions throughout pregnancy. The figure was created by the authors based on the literature reviewed in this article.

Beyond macronutrient composition, functional dietary components including prebiotics, polyphenols, omega-3 fatty acids, and fermented foods may further modulate microbial diversity, inflammatory pathways, and microbiota-derived metabolite production during pregnancy [48,49,50]. The mechanistic interactions between maternal diet, gut microbiota, and fetal programming are summarized in Figure 1.

These molecular interactions provide the mechanistic basis for understanding how fermented foods and functional dietary components modulate maternal gut microbiota, as discussed in the following sections.

## 4. Fermented Foods as Modulators of the Gut Microbiome

Fermented foods are increasingly recognized as functional dietary components capable of modulating gut microbiota composition and metabolic activity [46,47,48]. These foods include fermented dairy products, vegetables, cereals, soy-based products, and fermented beverages, all of which undergo biochemical transformations during fermentation that generate diverse bioactive compounds [15,51,52,53,54].

Beyond live microorganisms, fermented foods contain fermentation-derived metabolites including short-chain fatty acids (SCFAs), bioactive peptides, bacteriocins, exopolysaccharides, and phenolic metabolites that may contribute to gut homeostasis and immune regulation [15,17,55]. Current evidence suggests that the health effects of fermented foods are mediated through complex microbiota–host interactions rather than solely through probiotic microorganisms [56].

Consumption of fermented foods has been associated with increased microbial diversity and enrichment of beneficial taxa, including *Bifidobacterium*, *Lactobacillus*, *Akkermansia*, and butyrate-producing bacteria. Fermented dairy products, vegetables, and soy-based foods have been reported to promote beneficial microbial communities while suppressing potential pathobionts [16,57,58,59].

One of the principal mechanisms involves enhanced short-chain fatty acid (SCFA) production, which contributes to intestinal barrier integrity, immune regulation, and metabolic homeostasis through microbiota–host signaling pathways, as discussed in Section 2 [16,60].

Fermentation-derived metabolites may exert antioxidant and anti-inflammatory effects through modulation of intracellular signaling pathways [61,62,63]. In addition, bacteriocins and organic acids produced by lactic acid bacteria may inhibit enteric pathogens and support microbial homeostasis [51,54].

Emerging evidence also suggests that fermented foods have been associated with the microbiota–gut–brain axis through microbial metabolites involved in neuroimmune and neuroendocrine signaling [64]. However, current evidence is derived primarily from observational or short-term intervention studies, limiting causal inference and highlighting the need for well-designed long-term clinical trials during pregnancy.

Evidence specifically from pregnancy suggests that fermented foods may beneficially modulate the maternal gut microbiota, although the available data remain limited [6,65]. Regular consumption of fermented dairy products, particularly yogurt and kefir, has been associated with increased abundance of beneficial bacteria and improved microbial diversity [58,59,66,67]. Fermented foods may also enhance short-chain fatty acid production and contribute to the maintenance of intestinal barrier integrity and immune homeostasis through microbiota-mediated mechanisms [16,51,54,60]. These microbiota-related changes may support maternal metabolic homeostasis and immune tolerance during pregnancy [6,65,68]. However, not all studies have demonstrated significant improvements in gut microbial diversity or composition following fermented food consumption. Reported effects appear to vary according to the type of fermented food, microbial viability, habitual dietary patterns, baseline microbiota composition, and study design. Therefore, further longitudinal studies and well-designed randomized controlled trials are needed to clarify the specific effects of fermented foods on the maternal gut microbiota throughout gestation [65,68].

Beyond their direct effects on maternal gut microbiota, fermented foods and other microbiota-targeted dietary strategies may also influence maternal–fetal communication and early-life microbial programming. These broader implications are discussed in the following sections.

**Figure 2:**
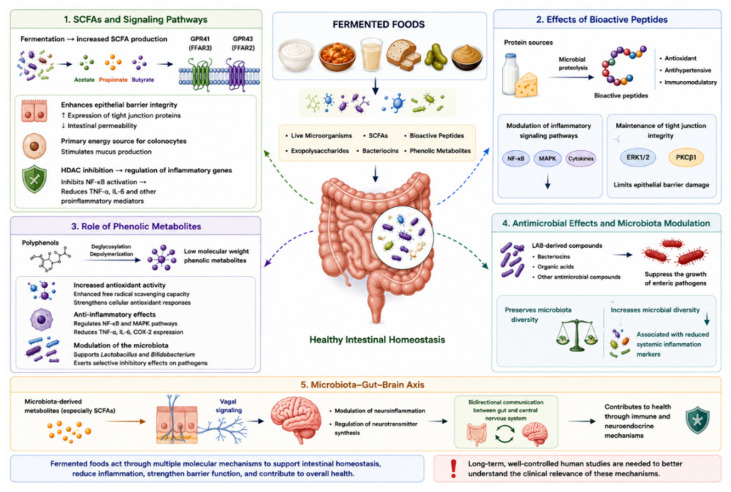
Molecular mechanisms through which fermented foods modulate gut microbiota composition and host physiology. Fermented foods provide live microorganisms and fermentation-derived bioactive metabolites that enhance microbial diversity, promote short-chain fatty acid (SCFA) production, strengthen intestinal barrier function, inhibit inflammatory signaling, and support immune and metabolic homeostasis. These effects involve modulation of G protein-coupled receptor (GPR)-mediated pathways, nuclear factor kappa B (NF-κB) signaling, and epithelial tight junction integrity. Abbreviations: SCFAs, short-chain fatty acids; GPR, G protein-coupled receptor; NF-κB, nuclear factor kappa B. Upward and downward arrows indicate increases and decreases, respectively, whereas dashed bidirectional arrows represent continuous interactions throughout pregnancy. The figure was created by the authors based on the literature reviewed in this article.

The molecular pathways through which fermented foods influence gut microbiota composition and host physiology are summarized in Figure 2.

## 5. Functional Nutrition and Microbiota-Targeted Dietary Components

### 5.1. Prebiotics and Selective Microbial Fermentation

Prebiotics are defined as non-digestible bioactive food components that are selectively utilized by gut microbiota and promote the growth of beneficial microorganisms [69]. Major prebiotics include inulin, fructooligosaccharides (FOS), galactooligosaccharides (GOS), and lactulose, which particularly stimulate the proliferation of *Bifidobacterium* and *Lactobacillus* species, thereby exerting bifidogenic effects [12,69].

Gut microbiota is highly influenced by environmental factors, particularly diet [70]. During pregnancy, maternal dietary patterns may modulate gut microbiota composition and influence the production of microbial metabolites and immune-regulatory compounds transferred to the fetus [71]. Fermentation of prebiotics increases SCFA production, thereby supporting beneficial microbial activity and intestinal homeostasis during pregnancy [72,73,74,75,76].

The resulting SCFAs may contribute to immune regulation and intestinal homeostasis during pregnancy [76]. These findings suggest that prebiotics may serve as microbiota-accessible substrates capable of supporting beneficial microbial activity during pregnancy, although pregnancy-specific clinical evidence remains limited.

### 5.2. Probiotics and Host–Microbiota Signaling

According to the International Scientific Association for Probiotics and Prebiotics (ISAPP), probiotics are defined as live microorganisms that confer health benefits to the host when administered in adequate amounts [13]. Probiotic supplementation during pregnancy has been widely investigated for its potential role in supporting gut microbiota balance and alleviating gastrointestinal symptoms. In particular, Lactobacillus, *Bifidobacterium*, Streptococcus thermophilus, and *Saccharomyces boulardii* species have been associated with potential benefits for both maternal and infant health [65,77].

Experimental and mechanistic studies suggest that probiotics may contribute to gut homeostasis by producing antimicrobial compounds, reinforcing epithelial barrier integrity, promoting SCFA production, and modulating immune responses. However, evidence confirming these mechanisms in pregnant women remains limited [78,79,80].

Metagenomic and transcriptomic studies have associated gut dysbiosis in gestational diabetes mellitus with altered leptin expression and inflammatory responses. Although experimental studies suggest that probiotic interventions may reduce inflammatory cytokine levels and support immune-regulatory pathways, evidence from human pregnancy studies remains limited [81]. Randomized controlled trials in pregnant women have reported that *Lactobacillus* rhamnosus HN001 supplementation may reduce the risk of gestational diabetes, while combinations containing *Lactobacillus* acidophilus, *L. casei*, and *Bifidobacterium bifidum* has been reported to improve fasting glucose, insulin resistance, and lipid profiles [68,82].

Animal studies further indicate that maternal probiotic supplementation may exert long-term effects on offspring microbiota composition, glucose metabolism, and immune development [83,84]. These findings provide important mechanistic insights; however, they should not be directly extrapolated to human pregnancy without confirmation from well-designed clinical trials. In addition, evidence from systematic reviews and meta-analyses suggests that maternal probiotic intake may increase beneficial bacteria in breast milk and infant gut microbiota, while potentially influencing infant colic risk and early-life metabolic outcomes [85]. Nevertheless, randomized controlled trials evaluating probiotic supplementation during pregnancy have reported inconsistent findings across different strains and clinical outcomes [85,86].

However, these findings should be interpreted with caution because considerable heterogeneity exists among published studies. Differences in probiotic strains, dosage, timing and duration of supplementation, maternal characteristics, baseline gut microbiota composition, and clinical outcomes may partly explain the inconsistent results reported across randomized controlled trials [85,86,87].

Overall, while mechanistic and preclinical studies provide a strong biological rationale for probiotic supplementation during pregnancy, many proposed mechanisms have not yet been consistently confirmed in human intervention studies.

### 5.3. Synbiotics and Microbial Synergy

In 2019, the International Scientific Association for Probiotics and Prebiotics (ISAPP) redefined synbiotics as combinations of live microorganisms and selectively utilized substrates that confer health benefits to the host. Commonly used synbiotics typically combine probiotic strains such as *Lactobacillus* spp. and *Bifidobacterium* spp. with prebiotic substrates including fructooligosaccharides (FOS), galactooligosaccharides (GOS), and inulin [86].

Synbiotics may modulate gut microbiota composition and exert regulatory effects on metabolic, gastrointestinal, and immune functions [84]. Studies conducted in pregnant women suggest that synbiotic supplementation has been reported to improve certain clinical parameters associated with preeclampsia, including reductions in systolic and diastolic blood pressure, proteinuria, and serum creatinine levels [87].

Animal studies further indicate that maternal probiotic and synbiotic supplementation may exert long-term effects on offspring immune function and gut microbiota composition. Maternal synbiotic administration has been associated with increased IgA, sIgA, and IL-10 levels, along with reduced lipopolysaccharide concentrations and proinflammatory markers [88]. In addition, increased abundance of beneficial bacteria including Actinobacteria, *Bifidobacterium*, *Faecalibacterium*, *Roseburia*, and *Blautia* has been reported, accompanied by elevated acetate, butyrate, and other microbial metabolites [87,88].

Current evidence suggests that synbiotics may regulate gut microbiota composition, immune responses, and metabolic homeostasis through synergistic interactions between probiotic microorganisms and prebiotic substrates. Nevertheless, further well-designed randomized controlled trials are required to establish the long-term safety, efficacy, and clinical benefits of synbiotic supplementation during pregnancy.

Despite these promising findings, clinical evidence regarding probiotic and synbiotic supplementation during pregnancy remains inconsistent. The following section summarizes the major sources of heterogeneity that may explain these conflicting results.

### 5.4. Conflicting Evidence and Sources of Heterogeneity

Although probiotic and synbiotic supplementation during pregnancy has shown promising effects on maternal metabolic and immune health, findings remain inconsistent across clinical studies [24,68,74,79]. For example, supplementation with *Lactobacillus rhamnosus* HN001 was associated with a reduced prevalence of gestational diabetes mellitus (GDM), particularly among women of advanced maternal age or with previous GDM [13]. However, participants were recruited from an allergy-prevention cohort, which may limit the generalisability of these findings.

Ref. [89] reported no reduction in GDM incidence following supplementation with *Lactobacillus rhamnosus* GG and *Bifidobacterium animalis* subsp. *lactis* BB-12. Likewise, Lindsay et al. [90] and Pellonperä et al. [91] found no significant improvements in maternal glucose metabolism or GDM risk in women with overweight or obesity.

Evidence from randomized controlled trials evaluating probiotic and synbiotic supplementation during pregnancy remains inconsistent, despite several promising findings (Table 2). While supplementation with *Lactobacillus rhamnosus* HN001 was associated with a lower prevalence of gestational diabetes mellitus [83], several other trials reported no significant metabolic benefit (Table 2). These discrepancies are likely attributable to differences in probiotic strains, formulations, dosage, intervention timing, maternal characteristics, study populations, and outcome definitions [24,60,78,79,81]. Therefore, current evidence does not support universal recommendations for probiotic or synbiotic supplementation during pregnancy, highlighting the need for larger, well-designed trials using standardized protocols [68,74,79].

Overall, the available RCT evidence does not consistently support routine probiotic or synbiotic supplementation during pregnancy for metabolic or obstetric benefits. While several studies have reported improvements in glucose metabolism or GDM risk, others have shown no significant effects. The variability in findings is likely attributable to differences in probiotic strains, intervention protocols, participant characteristics, background diet, and outcome definitions. Consequently, current evidence supports a cautious and individualized interpretation rather than a universal recommendation.

### 5.5. Polyphenols and Microbial Metabolism

Polyphenols are plant-derived bioactive compounds with antioxidant, anti-inflammatory, and immunomodulatory properties [92]. Major classes include flavonoids, phenolic acids, lignans, stilbenes, and curcuminoids, which are widely found in fruits, vegetables, tea, cocoa, and grapes. Recent evidence suggests that polyphenols can modulate gut microbiota composition and support the growth of beneficial bacteria, thereby influencing metabolic and immune homeostasis [93].

A substantial proportion of dietary polyphenols reaches the colon without complete absorption in the small intestine, where they undergo microbial biotransformation by bacterial enzymes such as glycosidases and esterases [94]. These reactions generate smaller phenolic metabolites with improved bioavailability, which may promote the growth of beneficial bacteria, including *Akkermansia muciniphila*, *Bifidobacterium*, and *Lactobacillus*, thereby supporting immune and metabolic homeostasis [80].

Polyphenols may also exert prebiotic-like effects by promoting beneficial bacterial growth and microbial metabolic activity [95]. Experimental studies further suggest that maternal polyphenol intake may attenuate gut dysbiosis and support intestinal homeostasis and immune regulation [96,97]. In addition, polyphenol-rich diets have been reported to modulate immune regulation, lipid metabolism, and epigenetic pathways [43]. However, pregnancy-specific human intervention studies investigating polyphenol-mediated microbiota modulation remain limited.

Collectively, functional dietary components have been associated with pregnancy-associated gut microbiota remodeling by selectively enriching beneficial microbial taxa and modulating microbial metabolic activity [73,74,75,78,86]. These microbiota-mediated changes have been associated with improved intestinal barrier integrity, immune tolerance, and metabolic homeostasis, which are essential for healthy pregnancy [6,12,16,65]. In pregnant populations, prebiotics, probiotics, synbiotics, and polyphenols have been reported to increase the abundance of beneficial bacteria, including *Bifidobacterium* and *Lactobacillus*, while reducing inflammation-related microbial alterations [73,74,75,78,87,96]. However, current evidence remains heterogeneous because of differences in intervention type, dosage, treatment duration, and baseline maternal microbiota composition. Therefore, further well-designed longitudinal and randomized controlled studies are required to define the optimal microbiota-targeted nutritional strategies during pregnancy [6,65,86].

Taken together, these microbiota-targeted nutritional interventions may influence not only maternal metabolic health but also microbial transmission and immune programming in early life, thereby linking maternal nutrition with the maternal–infant microbiome axis.

**Figure 3:**
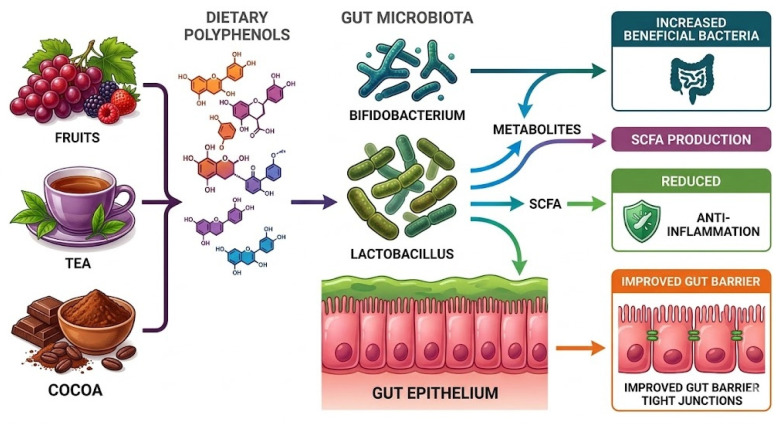
Polyphenol biotransformation and intracellular antioxidant and anti-inflammatory pathways. Dietary polyphenols are metabolized by the gut microbiota into bioactive metabolites that enhance short-chain fatty acid (SCFA) production, reduce oxidative stress, regulate inflammatory signaling pathways, and improve intestinal barrier integrity. These metabolites may also modulate immune responses and host metabolic homeostasis through microbiota-mediated molecular mechanisms. Abbreviation: SCFAs, short-chain fatty acids. The figure was created by the authors based on the literature reviewed in this article.

The proposed mechanisms underlying polyphenol biotransformation and its effects on gut microbiota and host signaling pathways are illustrated in Figure 3.

## 6. Microbiota and Maternal–Fetal Health Outcomes

### Maternal Metabolic Health During Pregnancy

Pregnancy-associated remodeling of the maternal gut microbiota has important implications for maternal metabolic health and pregnancy outcomes. Although these microbial adaptations are considered part of normal pregnancy, excessive alterations associated with maternal obesity, unhealthy dietary patterns, and gut dysbiosis may disrupt metabolic homeostasis and contribute to pregnancy-related complications [98,99,100].

Gestational diabetes mellitus (GDM) has been associated with reduced microbial diversity, altered short-chain fatty acid (SCFA) profiles, and impaired glucose homeostasis, suggesting that gut microbiota dysbiosis may contribute to disease development and potentially serve as an early predictive marker during pregnancy [101]. Similarly, maternal obesity and gut dysbiosis have been linked to an increased risk of preeclampsia through impaired intestinal barrier integrity, systemic inflammation, and reduced abundance of butyrate-producing bacteria, including *Coprococcus* [30,102].

Studies investigating microbiota-targeted interventions during pregnancy have produced heterogeneous findings. While some randomized controlled trials suggest that probiotic supplementation may improve gestational weight gain, insulin sensitivity, and the risk of preeclampsia [103], other studies have reported no significant effects on gestational diabetes or maternal metabolic outcomes [104]. Therefore, larger, well-designed longitudinal and randomized controlled studies are required to clarify the clinical efficacy of microbiota-targeted nutritional interventions during pregnancy.

Collectively, these findings suggest that maternal gut microbiota remodeling represents an important mechanistic link between maternal nutrition and pregnancy outcomes, providing a rationale for microbiota-targeted nutritional strategies discussed in the subsequent sections [6,10,99].

## 7. Maternal–Infant Microbiome Axis

The maternal–infant microbiome axis represents one of the central concepts linking maternal nutrition, gut microbiota, and offspring health [9,10,99]. Maternal dietary exposures influence microbial composition and metabolite production during pregnancy, which may subsequently affect fetal immune development, metabolic programming, microbial transmission, and early-life colonization [9,10,99,105,106]. Consequently, understanding this bidirectional axis provides an integrative framework connecting the molecular mechanisms discussed in the previous sections with long-term maternal and infant health outcomes [10,90,99].

### 7.1. Maternal Microbial Transmission

Although the fetal gastrointestinal system was long considered sterile, accumulating evidence suggests that maternal microbiota-derived metabolites and microbial products may influence fetal development during pregnancy [105]. Maternal gut, vaginal, and skin microbiota constitute the primary microbial exposure sources for the newborn during delivery, while emerging findings indicate that maternal microbial signals may also affect fetal physiology during the prenatal period.

Recent study has reported the detection of bacterial extracellular vesicles in amniotic fluid, suggesting a potential mechanism of maternal–fetal communication; however, these findings remain preliminary and require further validation [103]. However, because of the high contamination risk associated with low-biomass samples, it remains controversial whether these findings reflect true intrauterine colonization or transient prenatal microbial exposure [107].

Following birth, neonatal gut colonization is largely shaped by maternally derived microorganisms. Early colonizers include facultative anaerobic bacteria such as Escherichia coli and *Bifidobacterium*, followed by Bacteroides, Clostridium, and other anaerobic taxa [108,109]. Early-life microbiota development is considered a dynamic process involving developmental, transitional, and stabilization phases [110].

Breastfeeding and close maternal contact also play important roles in shaping infant gut microbiota. Retrograde bacterial transfer during breastfeeding and the proposed enteromammary pathway may facilitate the transfer of maternal gut bacteria into breast milk [111]. In addition, skin-to-skin contact after birth has been associated with increased microbial diversity and enhanced colonization of beneficial bacteria in infants [112,113].

Mode of delivery represents another major determinant of infant microbiota composition. Vaginally delivered infants generally exhibit higher abundance of *Bifidobacterium*, Bacteroides, and Escherichia species, whereas cesarean delivery has been associated with altered microbial diversity and differences in immune development [114].

Both animal and human studies indicate that maternal gut microbiota may influence fetal development and the gut–brain axis [46,115,116]. Alterations in maternal microbiota may affect fetal circulation through placental transfer of short-chain fatty acids (SCFAs), neurotransmitters, and inflammatory mediators [106]. In particular, acetate, propionate, and butyrate may regulate fetal immune development and neurodevelopment through GPR41/GPR43-mediated signaling pathways involved in inflammation control and immune tolerance [106,117].

Conversely, maternal dysbiosis-associated increases in intestinal permeability and lipopolysaccharide (LPS) translocation have been linked to systemic inflammation, hypothalamic–pituitary–adrenal (HPA) axis activation, and altered neurodevelopmental processes [116,118]. Maternal obesity-related metabolic and inflammatory alterations may also influence placental serotonin metabolism and lipid transport, potentially contributing to programming of the fetal gut–brain axis [119]. Nevertheless, the concept of in utero microbial colonization remains controversial because of methodological limitations and contamination concerns in low-biomass samples.

### 7.2. Human Milk and Bioactive Components

Human milk is not only a nutritional source but also a complex bioactive system that shapes infant gut microbiota composition and immune system development. Human milk contains human milk oligosaccharides (HMOs), milk-derived microorganisms, immunoglobulins, antimicrobial peptides, cytokines, and various growth factors that collectively support intestinal maturation, immune programming, and early-life microbial colonization [120,121].

HMOs are non-digestible complex carbohydrates that serve as selective prebiotic substrates, particularly for *Bifidobacterium* species. Fermentation of HMOs may contribute to the production of short-chain fatty acids (SCFAs), which contribute to lowering colonic pH, suppressing pathogens, and maintaining intestinal barrier integrity. HMOs have also been associated with modulation of Treg cell responses and promotion of immune tolerance [122].

HMO composition may influence microbial diversity and intestinal immunity. Variations associated with maternal secretor (FUT2) status have been linked to differences in infant gut microbiota composition, particularly increased abundance of beneficial bacteria such as Bacteroides, *Bifidobacterium*, and *Blautia*. In addition, HMOs may modulate immune responses by increasing Treg cells, type 2 innate lymphoid cells (ILC2), and serum IgA levels [123].

HMOs may further contribute to intestinal barrier maintenance by supporting Mucin 2 (MUC2)-mediated mucus production and regulating the expression of tight junction proteins including claudin, occludin, Zonula occludens-1 (ZO-1), and Junctional adhesion molecule-A (JAM-A), thereby reducing epithelial permeability [124]. These mechanisms may provide protection against intestinal inflammation and barrier dysfunction-related disorders.

Human milk microbiota is also influenced by maternally derived bacteria. Maternal–infant microbial transfer may occur through proposed enteromammary pathways and retrograde flow mechanisms, while migration of IgA+ plasma cells from Peyer’s patches to the mammary gland may contribute to shaping the immunoglobulin profile of breast milk [125].

Overall, HMOs and other bioactive components of human milk play central roles in early-life intestinal maturation and immune system programming through coordinated regulation of microbiota composition, microbial metabolite production, epithelial development, and immune responses [122].

### 7.3. Early-Life Microbial Colonization

Early-life microbial colonization plays a critical role in immune programming, metabolic homeostasis, and neurodevelopment during the “first 1000 days” of life [126]. Neonatal gut microbiota rapidly develops after birth and is strongly influenced by maternal microbiota, delivery mode, breastfeeding, and environmental exposures.

*Bifidobacterium* species, particularly *Bifidobacterium* longum subsp. infantis, B. bifidum, and B. breve, are dominant in the infant gut due to their ability to metabolize human milk oligosaccharides (HMOs) [127]. Breastfeeding and vertical microbial transmission support the colonization of these bacteria, whereas weaning may contribute to a more diverse, adult-like microbiota composition [119,125,126].

Beyond HMO metabolism, bifidobacteria contribute to anaerobic gut environment formation, microbial cross-feeding, and colonization resistance against potential pathogens [128]. Alterations in early-life microbiota composition and reduced microbial diversity have been associated with immune-mediated disorders including necrotizing enterocolitis, allergic diseases, asthma, and autoimmune conditions [129]. In particular, reductions in butyrate-producing bacteria may contribute to inflammatory processes [130].

The interaction between gut microbiota and the immune system is bidirectional. Early microbial exposure influences T-cell differentiation, Treg/Th1–Th2 balance, and inflammatory cytokine responses, thereby shaping neonatal immune development [124].

## 8. Clinical Implications and Translational Perspectives

Emerging evidence suggests that microbiota-targeted nutritional strategies may represent a promising approach for supporting maternal and fetal health during pregnancy. Dietary modulation of the maternal gut microbiota through fermented foods and functional dietary components has gained increasing attention because of its potential effects on metabolic regulation, immune tolerance, intestinal barrier integrity, and inflammatory homeostasis [6,10]. These mechanisms may be particularly relevant for the prevention or management of pregnancy-related complications such as gestational diabetes mellitus, excessive gestational weight gain, preeclampsia, and preterm birth [2].

From a clinical perspective, pregnant women should be encouraged to consume a balanced diet that includes safe, pasteurized fermented foods, such as yogurt and kefir, as part of an overall healthy dietary pattern, provided there are no medical contraindications. However, unpasteurized fermented dairy products and fermented foods associated with microbiological safety concerns should be avoided during pregnancy. At present, routine probiotic or synbiotic supplementation cannot be universally recommended, and decisions regarding supplementation should be individualized according to maternal health status, dietary intake, and clinical risk factors [74,77,79,131,132].

Fermented foods including yogurt, kefir, fermented vegetables, and traditional fermented products may contribute to microbial diversity and beneficial metabolite production. However, the clinical effects of fermented foods are likely influenced by substantial heterogeneity in microbial strains, fermentation methods, nutrient composition, and bioactive metabolite profiles [14,43]. Similarly, the efficacy of probiotics, prebiotics, synbiotics, and polyphenol-rich dietary interventions may depend on baseline maternal microbiota composition, dietary habits, host genetics, metabolic status, and environmental exposures [13,65,77,78,79,81].

Although randomized controlled trials have evaluated probiotic doses ranging from approximately 10^8^ to 10^10^ CFU/day, most interventions were initiated during the second trimester (approximately 14–20 weeks of gestation) and continued until delivery. However, substantial heterogeneity among probiotic strains, formulations, study populations, and intervention protocols precludes recommending an optimal dose, timing, or duration for routine clinical practice [13,65,77,78,79,81,82,83,84,85,86,87,88,89,90].

Despite growing mechanistic evidence, several translational limitations remain. Current human studies vary considerably in study design, intervention duration, dosage, and microbial characterization methods, limiting comparability across studies [47,55,108].

Current evidence indicates that probiotic supplementation is generally well tolerated in healthy pregnant women, with no consistent evidence of serious maternal or neonatal adverse events reported in randomized controlled trials. Nevertheless, caution is warranted in women with severe immunodeficiency, critical illness, or other high-risk clinical conditions, for whom safety data remain limited [76,83,85,89,90,91].

Advances in metagenomics, metabolomics, and precision nutrition approaches may facilitate the development of individualized microbiota-targeted dietary strategies during pregnancy [8,10]. Future longitudinal and randomized controlled studies integrating microbial, metabolic, immunological, and clinical outcomes are needed to clarify causal relationships and identify effective nutritional interventions capable of improving maternal and offspring health outcomes.

Although current evidence does not support universal recommendations for microbiota-targeted interventions during pregnancy, several practical considerations may assist clinicians in interpreting the available evidence and applying it in appropriate clinical contexts (Table 3).

Overall, microbiota-directed nutritional modulation represents a promising translational field with potential applications in personalized maternal nutrition and preventive obstetric care [2,5,8,83].

### Barriers to Clinical Implementation

Despite encouraging findings, several barriers currently limit the implementation of microbiota-targeted nutritional interventions in routine prenatal care. These include the lack of standardized probiotic formulations, uncertainty regarding optimal strain selection, dosage, timing, and duration of supplementation, considerable inter-individual variation in maternal gut microbiota composition, limited regulatory oversight of commercially available probiotic products, cost, product accessibility, and patient adherence [68,74,79]. Furthermore, the absence of consistent recommendations from major professional societies reflects the limited certainty of the current evidence base and highlights the need for large, well-designed multicentre randomized controlled trials before microbiota-targeted interventions can be routinely incorporated into obstetric practice [68,74,79,131].

Overall, microbiota-directed nutritional modulation represents a promising translational field with potential applications in personalized maternal nutrition and preventive obstetric care. However, current evidence supports an individualized rather than universal approach until stronger clinical evidence becomes available [2,6,10,74,79].

## 9. Limitations, Research Gaps, and Future Directions

Despite promising mechanistic findings, current evidence remains heterogeneous because of variations in study populations, microbial assessment techniques, probiotic strains, dietary assessment methods, intervention duration, and baseline microbiota composition. Inconsistencies between animal and human studies, together with the limited number of large-scale longitudinal and randomized controlled trials, further restrict the translation of current evidence into clinical practice. In addition, strain-specific probiotic effects and substantial interindividual variability in gut microbiota composition may partly explain the inconsistent findings reported across clinical studies. These limitations highlight important research gaps and underscore the need for standardized methodologies and well-designed longitudinal investigations.

## 10. Conclusions

This review highlights the maternal–infant microbiome axis as a central framework linking maternal diet, fermented foods, functional nutrition, microbial metabolism, and offspring health.

Maternal gut microbiota represents a dynamic interface between diet, metabolism, immune regulation, and fetal development during pregnancy. Emerging evidence suggests that fermented foods and functional dietary components, including prebiotics, probiotics, synbiotics, and polyphenols, may modulate gut microbiota composition and microbial metabolite production through mechanisms involving short-chain fatty acids, G protein-coupled receptor signaling, NF-κB regulation, histone deacetylase inhibition, and immune-modulatory pathways. These interactions may contribute to the maintenance of intestinal barrier integrity, metabolic homeostasis, and maternal immune tolerance, while also potentially influencing fetal programming and early-life microbiota development.

Although current mechanistic and clinical findings support the biological plausibility of microbiota-targeted nutritional strategies during pregnancy, substantial heterogeneity in study design, intervention characteristics, microbial assessment methods, and maternal metabolic status limits the establishment of standardized recommendations. Future research should increasingly focus on personalized nutrition approaches shaped by individual metabotypes, gut microbiota profiles, and maternal metabolic characteristics. The integration of multi-omics technologies, artificial intelligence-supported bioinformatic analyses, and advanced translational models may enable a more comprehensive molecular understanding of microbiota–host interactions. In addition, prospective longitudinal and randomized controlled studies are needed to evaluate the long-term effects of microbiota-targeted interventions during pregnancy and early life on both maternal and infant health outcomes. Current evidence supports biological plausibility; however, causality and long-term clinical efficacy remain insufficiently established.

## Figures and Tables

**Table 1:** Physiological and pathological maternal gut microbiota alterations during pregnancy.

Gestational Condition	Increased Taxa/Metabolites	Decreased Taxa/Metabolites	Major Molecular Pathways	Major Clinical Effects	Ref.
**Late Pregnancy (Physiological Adaptation)**	*Bifidobacterium*, Proteobacteria, acetate	Butyrate-producing bacteria (*Faecalibacterium prausnitzii*, *Roseburia*)	Altered SCFA metabolism; SCFA–GPR41/GPR43 signaling; metabolic adaptation pathways	Physiological insulin resistance, enhanced energy storage, fetal growth support	[26]
**Maternal Obesity**	Firmicutes, acetate/propionate	Bacteroidetes, butyrate producers	Increased intestinal permeability; LPS–TLR4/NF-κB signaling; metabolic endotoxemia	Barrier dysfunction, systemic inflammation, metabolic dysregulation	[27]
**GDM**	*Escherichia*, *Shigella*, LPS	*Bifidobacterium, Akkermansia*	LPS-mediated inflammation; impaired SCFA signaling; insulin resistance pathways	Metabolic inflammation, impaired glucose homeostasis, insulin resistance	[28]
**Preeclampsia**	Proteobacteria, TMAO, LPS	SCFA-producing bacteria, *Bifidobacterium*	TMAO-associated endothelial dysfunction; NF-κB activation; reduced butyrate signaling	Endothelial dysfunction, placental hypoperfusion, systemic inflammation	[29,30]

GDM, Gestational Diabetes Mellitus; LPS, Lipopolysaccharide; NF-κB, Nuclear Factor Kappa B; SCFA, Short-Chain Fatty Acid; TLR4, Toll-Like Receptor 4; TMAO, Trimethylamine N-oxide.

**Table 2:** Selected randomized controlled trials of probiotic/synbiotic supplementation during pregnancy and potential sources of heterogeneity.

Study	Intervention	Population	Main Findings	Potential Source(s) of Heterogeneity	Reference
**Wickens et al.**	*Lactobacillus rhamnosus* HN001 (6 × 10^9^ CFU/day) from 14–16 weeks’ gestation	Pregnant women with a personal or partner history of allergic disease	Reduced GDM prevalence, particularly in women of advanced maternal age or with previous GDM	Allergy-prevention cohort; secondary metabolic outcome; findings varied according to GDM diagnostic criteria	[13]
**Lindsay et al.**	*Lactobacillus salivarius* UCC118 for 4 weeks	Pregnant women with obesity	No significant improvement in maternal glucose metabolism or pregnancy outcomes	Short intervention duration; late initiation; obese population	[90]
**Callaway et al. (SPRING trial)**	*L. rhamnosus GG* + *B. animalis* subsp. *lactis* BB-12	Pregnant women with overweight or obesity	No reduction in GDM incidence; reduced excessive gestational weight gain	High-risk population; strain-specific effects; different primary outcomes	[89]
**Pellonperä et al.**	Probiotics (*L. rhamnosus* HN001 + *B. animalis* subsp. *lactis* 420) with or without fish oil	Pregnant women with overweight or obesity	No significant reduction in GDM incidence or improvement in glucose metabolism	Four-arm factorial design; overweight/obese population; combined intervention	[91]

**Abbreviations:** GDM, gestational diabetes mellitus; CFU, colony-forming units. The inconsistent findings across trials are likely attributable to differences in probiotic strains and formulations, study populations, intervention protocols, and outcome measures. Therefore, the results should not be interpreted as evidence of a uniform effect of probiotic or synbiotic supplementation during pregnancy.

**Table 3:** Practical Considerations for Microbiota-Targeted Nutritional Interventions During Pregnancy.

Topic	Current Evidence	Clinical Implication
**Dietary pattern**	Balanced diet including safe, pasteurized fermented foods may support gut microbiota diversity [14,51,73,133]	Encourage yogurt, kefir and other pasteurized fermented foods as part of a healthy diet.
**Probiotic supplementation**	Evidence remains inconsistent because of strain-specific effects and study heterogeneity [68,74,79]	Routine supplementation cannot currently be recommended for all pregnant women.
**Dose and timing**	Most RCTs evaluated 10^8^–10^10^ CFU/day, initiated at 14–20 weeks’ gestation until delivery [13,65,77,78,79,81,82,83,84,85,86,87,88,89,90]	No optimal dose, strain or duration has been established.
**Safety**	Generally well tolerated in healthy pregnant women; evidence in high-risk populations remains limited [68,74,79]	Use cautiously in immunocompromised or critically ill patients.
**Future implementation**	Precision nutrition and microbiome-guided interventions are promising but require further validation [131,134]	Individualized approaches may become feasible as evidence evolves.

## Data Availability

No new data were created or analyzed in this study. Data sharing is not applicable to this article.

## Supplementary Materials

The following supporting information can be downloaded at: https://www.mdpi.com/article/10.3390/ijms27146488/s1.
