# Supplementary material for: Fermented Foods, Functional Nutrition, and Maternal Gut Microbiota During Pregnancy: Molecular Mechanisms and the Maternal–Infant Microbiome Axis
Source: Int J Mol Sci. 2026 Jul 21;27(14):6488. doi: 10.3390/ijms27146488 (PMC13411985; doi:10.3390/ijms27146488)
Supplement: Supplementary file 1 [file ijms-27-06488-s001.zip › ijms-4400172-supplementary.pdf]

Supplementary Table S1. Literature search strategy used for database searches

**Database**	**Search strategy**	**Search period**	**Filters**
PubMed	("pregnancy" OR "pregnant women") AND ("gut microbiota" OR microbiome) AND ("fermented foods" OR probiotics OR prebiotics OR synbiotics OR polyphenols OR "functional nutrition")	January 2015–March 2026*	English
Scopus	Same search strategy adapted to database indexing terms	January 2015–March 2026*	English
Web of Science	Same search strategy adapted to database indexing terms	January 2015–March 2026*	English

Inclusion criteria: Original human studies, randomized controlled trials, observational studies, systematic reviews, meta-analyses, and relevant mechanistic animal studies addressing maternal gut microbiota, fermented foods, functional nutrition, or pregnancy. **Exclusion criteria:** Conference abstracts, editorials, letters, duplicate publications, non-English articles, and studies not directly relevant to the scope of the review.
